# Game Day Time Lag, Behavioral Patterns, and Perceived Condition in Japanese Professional Baseball Players: A Cross-Sectional Study

**DOI:** 10.3390/sports14100417

**Published:** 2026-09-22

**Authors:** Kayo Yoshitani-Kuwabara, Yukina Yumen, Yumi Takayama, Miyoko Okuzono, Fumiaki Hanzawa, Narumi Nagai

**Affiliations:** Laboratory of Nutritional Physiology, Graduate School of Human Science and Environment, University of Hyogo, Hyogo 670-0092, Japan; yukina.yamamoto15@gmail.com (Y.Y.); hinatabokko0401@yahoo.co.jp (Y.T.); okuzono-miyoko@aimservices.co.jp (M.O.); hanzawa@shse.u-hyogo.ac.jp (F.H.); nagai@shse.u-hyogo.ac.jp (N.N.)

**Keywords:** game day time lag, professional baseball players, sleep timing, competition schedule, breakfast frequency

## Abstract

Alternating day- and night-game schedules may alter athletes’ daily behavioral patterns without time-zone travel. This study aimed to characterize the game day time lag (GDL) and within-player sleep–wake differences across day- and night-game schedules, as well as compare sleep–wake patterns, breakfast frequency, and perceived condition between schedule exposure groups. This cross-sectional study included 53 Japanese professional baseball players: 16 exposed to day- and night-game schedules (DN group) and 37 exposed only to day games (D group). Sleep–wake timing, meal frequency, and perceived condition were assessed using MCTQ-based items, self-reporting, and eight 100 mm visual analog scales. The GDL was defined as the absolute difference in self-reported midsleep between day- and night-game schedules for each player. The mean GDL in the DN group was 2.2 ± 0.5 h (95% CI: 1.9, 2.4 h). Within the DN group, sleep timing was later for night-game than day-game schedules, whereas sleep duration did not differ. Exploratory comparisons showed later sleep timing, lower breakfast frequency, and lower sleep satisfaction in the DN group than in the D group. The schedule exposure completely overlapped with the team status, limiting interpretation of these differences. These findings suggest the importance of considering differences in sleep timing related to the competition schedule when providing conditioning support to professional athletes.

## 1. Introduction

Athletes’ performance and well-being are shaped by physiological factors, behavioral routines, and psychological state. In elite sport, competition schedules may influence daily behaviors, including sleep timing, eating patterns, and recovery routines [1,2]. Adequate sleep is important for cognitive function, recovery, and physical performance [1]. Studies of elite team-sport athletes have shown that regular sleep–wake timing is associated with greater sleep efficiency, longer sleep duration, and lower night-to-night variability in sleep characteristics [3,4]. Performance timing may also vary according to chronotype [5], supporting interest in how externally imposed schedules interact with athletes’ daily timing. Recent experimental evidence further supports the importance of sleep and temporal factors regarding performance. A single night of sleep extension improved physical and cognitive performance, particularly during morning assessments, in physically active university students [6], while the cognitive effects of caffeine ingestion and mouth rinsing in adolescent male volleyball athletes differed according to the time of day [7]. A recent systematic review and meta-analysis of professional and semiprofessional team sport athletes also demonstrated that neuromuscular performance may vary according to the time of day, with some performance measures being more favorable in the late afternoon or early evening than in the morning [8].

Physiological circadian misalignment refers to a mismatch between endogenous circadian phase and behavioral or environmental timing and is most directly assessed with biological phase markers. Social jetlag (SJL), by contrast, is a behavioral proxy commonly quantified as the difference in midsleep between workdays and free days [9]. Day-to-day sleep timing variability describes variation across days regardless of its cause. SJL has been associated with sleep quality, metabolic outcomes, and cognitive performance [10,11,12]. In athletes, longer sleep duration and better sleep quality have been associated with more favorable mood and energy levels [13], whereas sleep deprivation has been shown to increase perceived exertion and fatigue [14].

Research on schedule-related sleep disruption in athletes has often focused on travel across time zones or late-evening competition. Recent real-world observations in elite contact sport athletes have also shown poorer sleep after competition than after noncompetition days, highlighting the potential influence of competition timing and post-competition recovery demands on athletes’ sleep [15]. Transmeridian travel may impair sleep and performance [16], and night matches have been associated with impaired sleep and recovery [17]. Although sleep regularity and duration among elite athletes have been examined [3,4], repeated shifts between day- and night-game schedules within the same time zone remain underexplored. Japanese professional baseball provides a relevant context because weekday night games typically begin at 18:00, whereas weekend day games typically begin at 14:00. Late game completion and subsequent post-game demands, including recovery activities, meals, transportation, and media obligations, may delay sleep opportunities and alter eating routines [15,17]. However, the effects of these repeated schedule-related shifts have received little research attention.

We therefore focused on the game day time lag (GDL) as an indicator of game-schedule-specific behavioral shifts in sleep timing. We hypothesized that players exposed to alternating day- and night-game schedules would exhibit a measurable GDL and, compared with players exposed only to day games, later sleep timing, lower breakfast frequency, and less favorable perceived condition. The aims of this study were (1) to characterize the GDL and within-player sleep–wake differences across day- and night-game schedules and (2) to compare sleep–wake patterns, breakfast frequency, and perceived condition between players exposed to alternating day- and night-game schedules and those exposed only to day games. In the present study, the GDL was operationally defined as the absolute within-player difference in self-reported midsleep between day- and night-game schedules; it was not intended as a direct measure of endogenous circadian phase.

## 2. Materials and Methods

### 2.1. Study Design

This observational cross-sectional study used a retrospective questionnaire-based assessment. Data were collected at a single time point in June 2023 during the regular competitive season. Participants were asked to recall their sleep–wake patterns, meal frequency, and perceived condition during the preceding month. The game schedule during the one-month recall period was consistent with the usual distribution of day and night games during the regular season. The study procedure consisted of participant recruitment, questionnaire administration, schedule exposure classification, GDL calculation, and statistical comparisons of sleep–wake patterns, meal frequency, and perceived condition.

### 2.2. Participants

Players were eligible if they were active members of either the first or second team of Team A at the time of the survey and had 1–10 years of professional baseball experience. All eligible Japanese players were invited to participate in the study. No additional recruitment methods were used. Players were excluded from the final analysis if they had been unable to participate in regular training during the 2 weeks preceding the survey because of injury, rehabilitation, or other reasons, or if a recent transfer between the first and second teams had markedly changed their lifestyle patterns.

Of the 68 players assessed for eligibility to participate, 10 did not participate because they were foreign players, were hospitalized, or were unavailable for other reasons. The remaining 58 players provided written informed consent and completed the survey. Five players were excluded before the final analysis: one had been unable to participate in regular training during the 2 weeks preceding the survey, and four had recently transferred between the first and second teams, resulting in marked changes in their lifestyle patterns. Thus, 53 players were included in the final analysis (Figure 1A).

The study protocol was approved by the Research Committee of the School of Human Science and Environment, University of Hyogo (Approval No. 312; 24 May 2022), and was conducted in accordance with the Declaration of Helsinki. Response sheets carried study codes rather than names, and the primary dataset was managed securely by the principal investigator.

### 2.3. Data Collection

Data were collected once using a self-administered questionnaire, athlete records, and official team game logs. Questionnaires were distributed individually with completion instructions, completed without names and identified only using study codes, and returned in a designated collection box. All participants completed the questionnaires under standardized conditions in the same designated room during the morning before training. All questionnaires from the 53 players included in the final analysis were returned on the data collection day. A schematic overview of the study protocol, including schedule exposure classification, questionnaire administration, outcome assessment, GDL calculation, and statistical comparisons, is presented in Figure 1B.

### 2.4. Participant Characteristics and Schedule Exposure Classification

Total years of baseball playing experience, height, weight, and living arrangement were obtained from the self-administered questionnaire. Body mass index (BMI; kg/m^2^) was calculated as weight (kg) divided by height squared (m^2^). Current age, age at team entry, years of professional experience, and playing position were obtained from player records, and schedule information was obtained from official game logs.

Schedule exposure was determined by team status. First-team players, who typically played weekend day games starting at approximately 14:00 and weekday night games starting at approximately 18:00 from Tuesday to Friday, constituted the mixed day/night schedule exposure group (DN). Second-team players, who typically played day games starting at approximately 14:00 from Tuesday to Sunday, constituted the day-only schedule exposure group (D). Thus, the schedule group completely overlapped with the first- versus second-team status. Between-group analyses were treated as exploratory because differences could reflect the team status, training, travel, playing opportunities, or other contextual factors rather than game timing alone.

### 2.5. Sleep–Wake Assessment and Game Day Time Lag

Sleep–wake timing was assessed retrospectively using MCTQ-based questions [18]. Participants were asked to recall their usual sleep–wake patterns during the preceding month and to report their average bedtime, wake-up time, and sleep latency separately for day-game days, night-game days, and days off, as applicable. Sleep duration and midsleep were derived from the reported times for each schedule condition.

The GDL was calculated separately for each DN participant using the following equation:

GDL = |midsleep on night-game days − midsleep on day-game days|.

Individual GDL values were subsequently summarized as the mean ± SD for the DN group. Clock times crossing midnight were handled on a continuous time scale. The GDL represents schedule-related behavioral sleep-timing displacement and should not be interpreted as a direct measure of endogenous circadian phase or physiological circadian disruption.

### 2.6. Meal Frequency Assessment

Breakfast frequency was designated a priori as the primary dietary outcome because previous work has linked social jetlag with dietary patterns [19]. Participants reported whether they usually consumed breakfast, lunch, and dinner on day-game days, night-game days, and days off. Responses were combined according to each group’s weekly schedule to calculate meal frequencies in times per week.

### 2.7. Subjective Condition Assessment

The questionnaire included eight visual analog scale (VAS) items concerning the preceding month: overall condition, physical heaviness, fatigue, sleep satisfaction, vitality/motivation, physical sharpness, emotional stability, and appetite [20]. Each item was assessed using a 100 mm horizontal VAS, with 0 and 100 mm indicating the least and most favorable conditions, respectively. For physical heaviness and fatigue, higher scores indicated less heaviness and less fatigue; no reverse coding was applied. Participants marked the point that best represented their condition, and one examiner measured the distance from the 0 mm anchor using the same ruler [21,22]. Scores were interpreted as self-reported condition indicators rather than objective psychological or performance measures.

### 2.8. Statistical Analysis

Data are presented as the mean ± SD or *n* (%). No item-level data were missing for the variables included in the analyses. Consequently, no imputation was performed. Normality was assessed using the Shapiro–Wilk test. The DN and D groups were compared using independent-sample *t* tests, Welch’s *t* tests, Mann–Whitney U tests, or chi-square/Fisher’s exact tests, as appropriate.

Sleep–wake variables across night-game, day-game, and off-day schedules in the DN group were analyzed using one-way repeated-measures analysis of variance. Sphericity was assessed using Mauchly’s test, and the Greenhouse–Geisser correction was applied when necessary. Significant main effects were then subject to Bonferroni-adjusted pairwise comparisons. Day-game and off-day schedules in the D group were compared using paired *t* tests. Times after midnight were treated as times on the following day.

Effect sizes are reported as partial η^2^ for repeated-measures analyses, Cohen’s dz for paired *t* tests, Cohen’s d for independent-sample t tests, and rank-biserial correlation (rrb) for Mann–Whitney U tests. The corresponding 95% CIs are reported for Cohen’s d and dz. The 95% CIs for rank-biserial correlations were calculated using percentile bootstrapping with 10,000 resamples. No adjustment was applied across different outcome variables; these results were interpreted as exploratory. All tests were two-tailed, with *p* < 0.05 denoting statistical significance. Analyses were performed using SPSS Statistics version 24.0 (IBM Corp., Tokyo, Japan).

The required sample size was calculated using G*Power version 3.1.9.7 [23]. Because no previous effect estimate was available for the GDL, breakfast frequency was used for the calculation based on its reported association with social jetlag [19]. With an effect size of 0.8, α = 0.05, and 80% power, the target sample size was 54; the final sample included 53 players.

## 3. Results

### 3.1. Participant Characteristics

Of the 58 consenting players, five met the exclusion criteria, leaving 53 players for analysis. Participant characteristics are presented in Table 1. The analysis included 53 players (DN, *n* = 16; D, *n* = 37). The groups did not differ significantly in age, baseball career, height, living arrangement, or playing position. Weight and BMI were lower in the DN group than in the D group (both *p* = 0.020).

### 3.2. Sleep–Wake Patterns and GDL

Between-group and within-group comparisons are presented in Table 2 and Table 3, respectively. Within the DN group, bedtime, wake-up time, midsleep, and sleep duration differed significantly across the day-game, night-game, and off-day schedules (all overall *p* < 0.001; partial *η*^2^ = 0.506–0.849; Table 3). Bedtime and midsleep were later on night-game days than on day-game days and days off. Wake-up time was later on night-game days and days off than on day-game days. Sleep duration was longer on days off than for both game schedules, whereas it did not differ significantly between night- and day-game days. Pairwise *p*-values were Bonferroni-adjusted.

The mean GDL in the DN group was 2.2 ± 0.5 h (95% CI: 1.9, 2.4 h), representing the average magnitude of the within-player difference in midsleep between day- and night-game schedules. The distribution of individual GDL values among the 16 DN players is shown in Figure 2. The GDL could not be calculated in the D group because night-game observations were unavailable.

Within the D group, bedtime, wake-up time, midsleep, and sleep duration differed significantly between day-game days and days off (all *p* < 0.001; Table 3). Bedtime, wake-up time, and midsleep were later, and sleep duration was longer on days off than on day-game days.

In exploratory between-group comparisons on day-game days, the DN group had a later bedtime (23:28 vs. 23:05, *p* = 0.037, rank-biserial correlation = 0.345, 95% CI: 0.010, 0.654), wake-up time (07:12 vs. 06:29, *p* < 0.001, Cohen’s *d* = 1.286, 95% CI: 0.643, 1.917), and midsleep (03:20 vs. 02:47, *p* < 0.001, Cohen’s *d* = 1.143, 95% CI: 0.512, 1.765) than the D group (Table 2). Sleep duration did not differ significantly between the groups (7.6 ± 0.8 vs. 7.4 ± 0.7 h, *p* = 0.254, Cohen’s *d* = 0.345, 95% CI: −0.247, 0.934). On days off, no significant between-group differences were observed regarding bedtime, wake-up time, midsleep, or sleep duration (all *p* ≥ 0.175; Table 2).

### 3.3. Meal Frequency

In exploratory between-group comparisons, weekly breakfast frequency was lower in the DN group than in the D group (3.9 ± 2.0 vs. 6.4 ± 1.2 times/week, *p* = 0.002, rank-biserial correlation = −0.490, 95% CI: −0.794, −0.155). No significant between-group differences were observed in weekly lunch frequency (6.4 ± 1.4 vs. 6.8 ± 0.4 times/week, *p* = 0.842, rank-biserial correlation = 0.025, 95% CI: −0.235, 0.267) or dinner frequency (7.0 ± 0.0 vs. 7.0 ± 0.0 times/week, *p* = 1.000, rank-biserial correlation = 0.000, 95% CI: 0.000, 0.000; Table 2).

### 3.4. Perceived Condition (VAS Scores)

Table 4 presents the eight self-reported condition scores. Sleep satisfaction was lower in the DN group than in the D group (55.7 ± 19.9 vs. 67.5 ± 17.5 mm, *p* = 0.035, Cohen’s *d* = −0.648, 95% CI: −1.244, −0.045). No significant between-group differences were observed for the other seven VAS outcomes.

## 4. Discussion

### 4.1. Principal Findings

This study characterized the GDL as a within-player measure of game-schedule-related sleep-timing displacement and explored differences in behavioral patterns between schedule groups. The principal finding was a mean GDL of 2.2 ± 0.5 h (95% CI: 1.9, 2.4 h) in the DN group. This finding documents a midsleep displacement of more than 2 h between day- and night-game schedules without time zone travel. Within the DN group, sleep timing was later on night-game than on day-game schedules, whereas sleep duration did not differ between the two game schedules. Sleep duration was longer on days off than on either game schedule.

In the exploratory between-group comparisons, the DN group had a lower weekly breakfast frequency and lower sleep satisfaction than the D group. The other seven VAS outcomes did not differ between the groups. These findings show differences in sleep timing and breakfast frequency, as well as an isolated difference in sleep satisfaction, but do not establish causal effects of game schedules or individual-level associations with the GDL.

The between-group findings should be interpreted cautiously because the schedule group completely overlapped with the team status. Body weight and BMI were also lower in the DN group, indicating that the groups were not fully comparable at baseline. Although these differences may not directly explain the findings, their potential influences on sleep timing, breakfast frequency, and sleep satisfaction cannot be excluded.

### 4.2. Sleep Timing and Game Schedules

The later sleep timing observed after night games is consistent with previous reports of delayed or altered sleep following evening competition [20]. The mean GDL of 2.2 h indicates that players adjusted their sleep timing by more than 2 h between day- and night-game schedules. From a practical perspective, a displacement of this magnitude may present challenges for maintaining consistent sleep and recovery routines across changing game schedules. However, sleep duration did not differ significantly between night- and day-game schedules, and the effects of the GDL on recovery and performance were not directly assessed.

Post-game arousal, a delayed return home, recovery activities, evening meals, light exposure, and other scheduling constraints could plausibly contribute to later sleep timing, but these factors were not measured. Moreover, the GDL was quantified based on self-reported behavioral sleep-timing displacement rather than physiological circadian phase; objective sleep and circadian measures are therefore required to determine whether the observed pattern reflects circadian misalignment, changes in recovery opportunities, or both.

### 4.3. Meal Frequency and Perceived Condition

Breakfast frequency was lower in the DN group than in the D group, with a moderate between-group effect size. This finding is consistent with reports linking later or less regular sleep timing with dietary patterns in non-athlete populations [19,24]. However, the difference cannot be attributed specifically to the GDL because the schedule group completely overlapped with the team status. Appetite, meal availability, post-game nutrition, travel, training schedules, and living arrangements may have also contributed. Potential effects on energy availability, recovery, readiness, and performance were not assessed and should be examined in prospective studies.

Among the eight VAS outcomes, only sleep satisfaction was lower in the DN group than in the D group, with a moderate effect size (Cohen’s *d* = −0.648). This finding does not indicate consistently poorer perceived condition across all domains in the DN group. The wide SDs suggest considerable individual variability. Moreover, because the VAS comparisons were exploratory and the groups differed in team status and baseline characteristics, the difference in sleep satisfaction should be interpreted cautiously and cannot be attributed solely to game schedule exposure.

### 4.4. Interpretation and Practical Implications

GDL may provide a simple, sport-specific operational indicator of the extent to which an athlete’s reported midsleep shifts between different competition schedules. Unlike SJL, which contrasts work and free days, GDL contrasts day- and night-game schedules. Its within-player definition is a strength because it focuses on differences between schedule conditions. However, self-reported midsleep does not directly measure circadian phase, sleep quality, or physiological disruption. Thresholds for a practically meaningful GDL and its applicability to other sports, competition calendars, and cultural settings remain to be established.

The present findings suggest the potential value of monitoring schedule-related changes in sleep timing and opportunities for meals. However, they do not demonstrate the effectiveness of any specific intervention. Candidate strategies for future testing include structured post-game recovery, planned access to meals or snacks, individualized sleep-timing support, and consideration of chronotype. Any changes to team scheduling, recovery routines, or conditioning practices should be evaluated prospectively for their feasibility and effects on sleep, nutrition, recovery, and performance.

### 4.5. Strengths and Limitations

The strength of this study is its within-player assessment of schedule-related shifts in sleep timing in a professional sports setting. Rather than relying solely on the group mean, we presented the distribution of individual GDL values to demonstrate variability among players.

Several limitations should be considered. First, the schedule group completely overlapped with the team status: DN players belonged to the first team, whereas D players belonged to the second team. The groups also differed in body weight and BMI. Therefore, the between-group comparisons cannot separate the influence of game schedules from those of team status, anthropometric characteristics, competitive level, training load, travel, playing opportunities, recovery resources, or other lifestyle factors.

Second, the cross-sectional design does not allow for causal inference. Third, sleep timing and meal frequency over the preceding month were assessed retrospectively via self-reporting and may have been affected by recall or reporting bias. Fourth, the DN group was small, and the final sample of 53 players was one participant below the planned target. Therefore, the statistical power and precision, particularly for small-to-moderate effects, may have been limited. Because several outcomes were examined, the exploratory between-group findings should also be interpreted cautiously.

Fifth, associations between individual GDL values and breakfast frequency, sleep satisfaction, or other outcomes were not examined because the DN group included only 16 players. Therefore, the present findings do not demonstrate individual-level associations between the GDL and these outcomes. Sixth, chronotype, naps, caffeine and alcohol intake, evening screen use and light exposure, post-game nutrition, game participation, travel, and training load were not assessed. Finally, all participants were recruited from a single professional baseball organization, which may limit the generalizability of the findings to other teams, sports, schedules, and cultural settings.

### 4.6. Future Directions

Future multicenter prospective studies should use repeated sleep diaries, actigraphy or validated wearable devices, circadian phase markers, and within-player observations across a competitive season. Further studies should also validate the GDL across sports and settings and examine its associations with nutrition, recovery, and performance in adequately powered samples.

## 5. Conclusions

This study characterized the GDL and within-player sleep–wake differences across day- and night-game schedules in Japanese professional baseball players. The mean self-reported GDL in the DN group was approximately 2.2 h. These findings suggest substantial midsleep displacement within players between day- and night-game schedules, even without time-zone travel.

The GDL may provide a framework for describing schedule-related shifts in sleep timing, but it does not directly measure physiological circadian disruption. Further studies using objective sleep measures and intervention designs are needed to determine whether the GDL and game schedules are associated with athletes’ daily behaviors and perceived conditions.

## Figures and Tables

**Figure 1:**
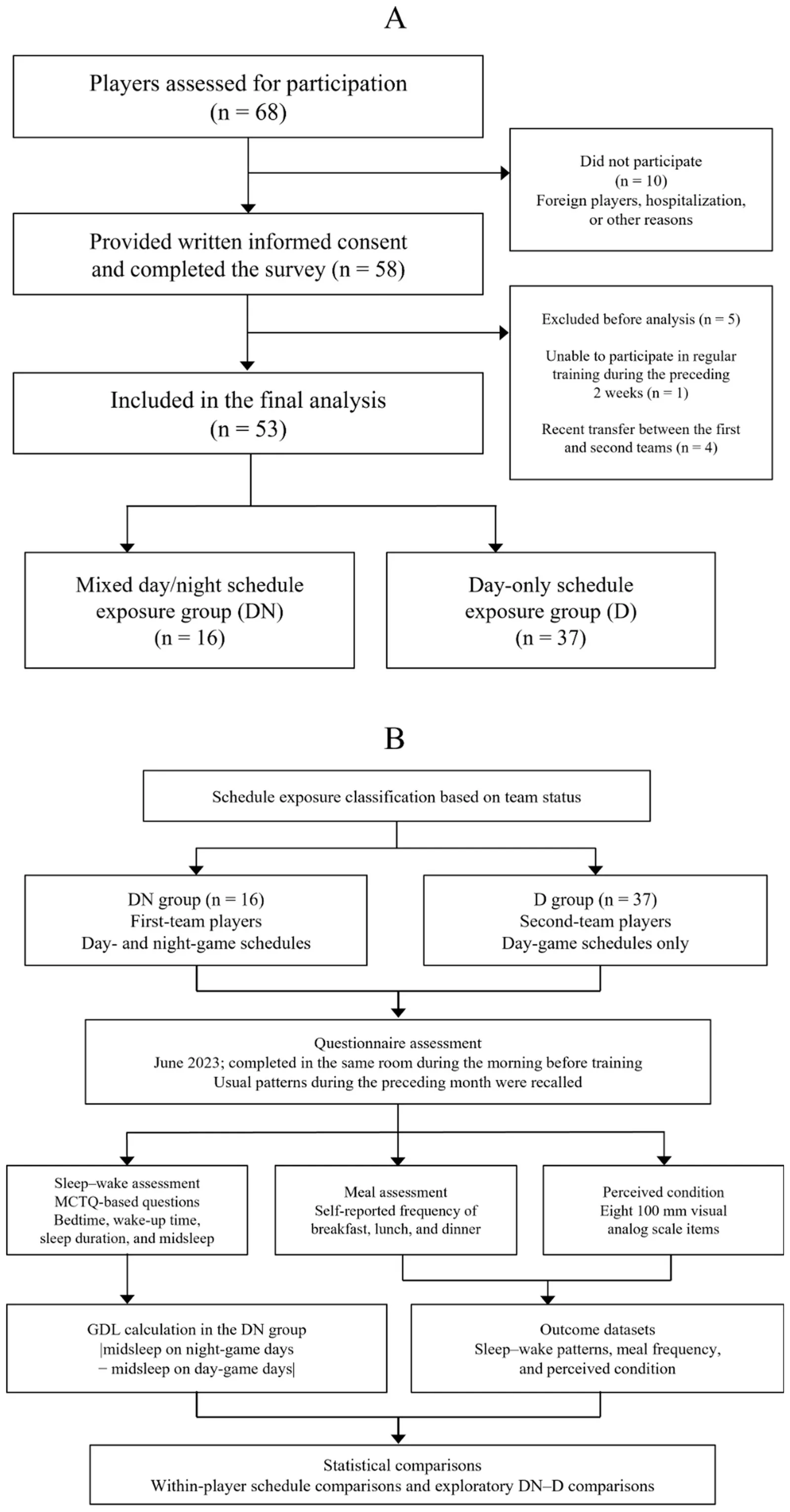
Participant flow and study procedures: (**A**) participant recruitment and selection; (**B**) schedule exposure classification, assessments, and analyses. DN, mixed day- and night-game schedule group; D, day-game schedule group; MCTQ, Munich ChronoType Questionnaire; GDL, game day time lag.

**Figure 2:**
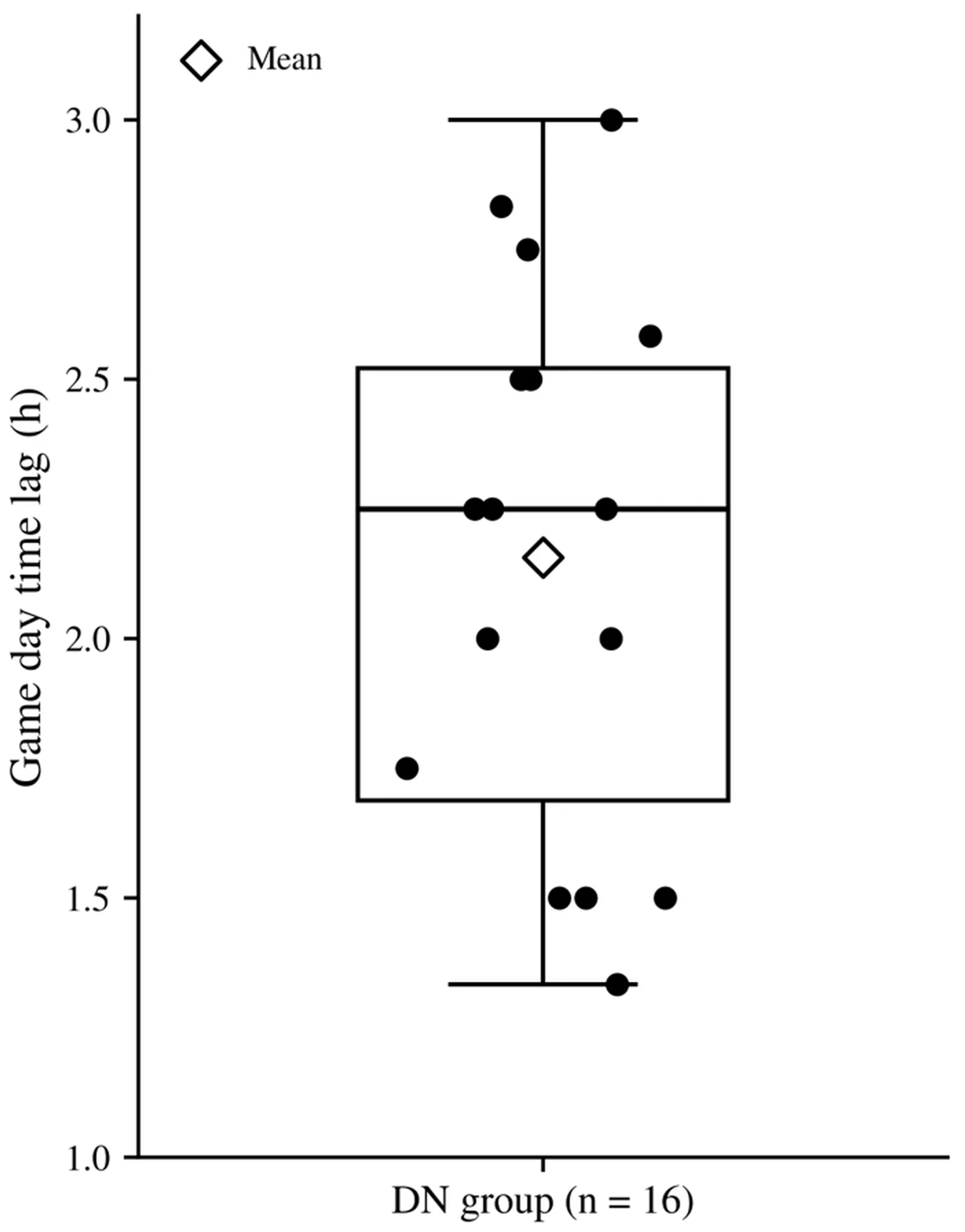
Distribution of individual GDL values in the DN group (*n* = 16). Filled circles represent individual participants; the box, median and interquartile range; whiskers, range; and open diamond, mean. GDL, game day time lag; DN, mixed day- and night-game schedule.

**Table 1:** Participant characteristics by schedule exposure group.

	DN			D			*p*-Value
	(*n* = 16)			(*n* = 37)			
Age (y)	26.1	±	4.0	24.5	±	3.8	0.140 ^†^
Baseball playing experience (y)	17.6	±	4.3	17.3	±	4.3	0.840 ^†^
Height (cm)	178.0	±	5.0	180.1	±	5.2	0.190 ^†^
Weight (kg)	80.2	±	6.9	85.3	±	7.3	0.020 ^†^
Body mass index (kg/m^2^)	25.3	±	1.3	26.3	±	1.5	0.020 ^†^
Living arrangement							
Dormitory	5	(31.3)		16	(43.2)		0.710 ^‡^
Living alone	3	(18.8)		6	(16.2)		
Living with family	8	(50.0)		15	(40.5)		
Position							
Pitcher	7	(43.8)		20	(54.1)		0.490 ^‡^
Fielder *	9	(56.3)		17	(45.9)		

Notes: Values are presented as mean ± SD or *n* (%). DN, mixed day- and night-game schedule group; D, day-game schedule group. Schedule group corresponded to team status. ^†^ Independent-sample *t* test; ^‡^ chi-square test. * Fielders included catchers, infielders, and outfielders.

**Table 2:** Exploratory comparisons of sleep–wake patterns and meal frequency between the DN and D groups.

Panel A. Day-game days				
Variable	DN Group	D Group	*p*-Value	Effect Size (95% CI)
Bedtime	23:28 ± 0:38	23:05 ± 0:38	0.037 ^†^	*r_rb_* = 0.345 (0.010, 0.654)
Wake-up time	7:12 ± 0:28	6:29 ± 0:29	<0.001 ^‡^	*d* = 1.286 (0.643, 1.917)
Midsleep point	3:20 ± 0:24	2:47 ± 0:29	<0.001 ^‡^	*d* = 1.143 (0.512, 1.765)
Sleep duration (h)	7.6 ± 0.8	7.4 ± 0.7	0.254 ^‡^	*d* = 0.345 (−0.247, 0.934)
Panel B. Days off				
Variable	DN Group	D Group	*p*-Value	Effect Size (95% CI)
Bedtime	0:05 ± 1:06	23:57 ± 0:47	0.383 ^§^	*d* = 0.305 (−0.286, 0.893)
Wake-up time	9:14 ± 0:52	8:56 ± 1:11	0.191 ^‡^	*d* = 0.396 (−0.197, 0.986)
Midsleep	4:39 ± 0:54	4:27 ± 0:50	0.175 ^‡^	*d* = 0.412 (−0.182, 1.002)
Sleep duration (h)	9.1 ± 0.8	8.9 ± 1.1	0.362 ^†^	*r_rb_* = 0.155 (−0.159, 0.470)
Panel C. Weekly meal frequency				
Variable (times/week)	DN Group	D Group	*p*-Value	Effect Size (95% CI)
Breakfast	3.9 ± 2.0	6.4 ± 1.2	0.002 ^†^	*r_rb_* = −0.490 (−0.794, −0.155)
Lunch	6.4 ± 1.4	6.8 ± 0.4	0.842 ^†^	*r_rb_* = 0.025 (−0.235, 0.267)
Dinner	7.0 ± 0.0	7.0 ± 0.0	1.000 ^†^	*r_rb_* = 0.000 (0.000, 0.000)

Notes: Values are presented as mean ± SD. ^†^ Mann–Whitney U test; ^‡^ independent-sample *t* test; ^§^ Welch’s *t* test. CI, confidence interval; *d*, Cohen’s d; *r_rb_*, rank-biserial correlation coefficient. DN, mixed day- and night-game schedule group; D, day-game schedule group.

**Table 3:** Within-group comparisons of sleep–wake patterns across game schedules.

Panel A. DN group (*n* = 16).					
Variable	Night-Game Days	Day-Game Days	Days Off	Overall *p*-Value	Partial *η*^2^
Bedtime	1:12 ± 0:46 ^a^	23:28 ± 0:38 ^b^	0:05 ± 1:06 ^c^	<0.001	0.702
Wake-up time	9:33 ± 1:00 ^a^	7:12 ± 0:28 ^b^	9:14 ± 0:52 ^a^	<0.001	0.849
Midsleep point	5:23 ± 0:48 ^a^	3:20 ± 0:24 ^b^	4:39 ± 0:54 ^c^	<0.001	0.849
Sleep duration (h)	8.3 ± 0.8 ^a^	7.6 ± 0.8 ^a^	9.1 ± 0.8 ^b^	<0.001	0.506
Panel B. D group (*n* = 37).					
Variable	Day-Game Days	Days Off	*p*-Value	Cohen’s *d_z_* (95% CI)	
Bedtime	23:05 ± 0:38	23:57 ± 0:47	<0.001	−1.326 (−1.765, −0.878)	
Wake-up time	6:29 ± 0:29	8:56 ± 1:10	<0.001	−2.304 (−2.920, −1.679)	
Midsleep point	2:47 ± 0:29	4:27 ± 0:50	<0.001	−2.392 (−3.025, −1.750)	
Sleep duration (h)	7.4 ± 0.7	8.9 ± 1.1	<0.001	−1.433 (−1.889, −0.967)	

Notes: Values are presented as mean ± SD. DN-group overall *p* values and partial *η*^2^ were obtained using one-way repeated-measures ANOVA; Different superscript letters (a, b, and c) indicate significant differences between conditions (Bonferroni-adjusted *p* < 0.05). D-group *p* values and Cohen’s *d_z_* were obtained using paired *t* tests. DN, mixed day- and night-game schedule group; D, day-only schedule group; SD, standard deviation; ANOVA, analysis of variance; *η*², partial eta squared; *d_z_*, Cohen’s *d_z_*; CI, confidence interval.

**Table 4:** Exploratory comparisons of perceived condition between the DN and D groups.

VAS Outcome	DN Group (*n* = 16)	D Group (*n* = 37)	*p*-Value	Cohen’s *d* (95% CI)
Overall condition	56.0 ± 22.1	60.0 ± 18.9	0.505	−0.201 (−0.788, 0.388)
Physical heaviness	50.3 ± 19.2	58.1 ± 20.8	0.210	−0.380 (−0.969, 0.213)
Fatigue	50.0 ± 15.6	48.3 ± 19.0	0.748	0.097 (−0.490, 0.683)
Sleep satisfaction	55.7 ± 19.9	67.5 ± 17.5	**0.035**	−0.648 (−1.244, −0.045)
Vitality/motivation	69.9 ± 19.4	75.4 ± 18.8	0.337	−0.290 (−0.878, 0.300)
Physical sharpness	56.1 ± 16.9	61.1 ± 15.0	0.281	−0.326 (−0.914, 0.265)
Emotional stability	62.3 ± 21.8	67.7 ± 19.9	0.378	−0.266 (−0.854, 0.324)
Appetite	71.0 ± 14.5	72.3 ± 17.0	0.796	−0.078 (−0.664, 0.509)

Notes: Values are presented as mean ± SD (mm). Scores range from 0 (least favorable condition) to 100 (most favorable condition); higher scores indicate better perceived condition. Between-group comparisons were performed using independent-sample t tests. Bold *p*-values indicate significant differences between the DN and D groups (*p* < 0.05). DN, mixed day- and night-game schedule group; D, day-game schedule group.

## Data Availability

The data presented in this study are available upon request from the corresponding author. The data are not publicly available due to privacy or ethical restrictions.

## Abbreviations

The following abbreviations are used in this manuscript:

GDL	Game day time lag
SJL	Social jetlag
BMI	Body mass index
DN group	Mixed day/night schedule exposure group
D group	Day-only schedule exposure group
MCTQ	Munich ChronoType Questionnaire
VAS	Visual analog scale
SD	Standard deviation
